# ASSOCIATION BETWEEN BREASTFEEDING AND OVERWEIGHT/OBESITY IN SCHOOLCHILDREN AGED 7-14 YEARS

**DOI:** 10.1590/1984-0462/2021/39/2020076

**Published:** 2021-02-24

**Authors:** Katia Jakovljevic Pudla Wagner, Camila Elizandra Rossi, Patrícia de Fragas Hinnig, Mariane de Almeida Alves, Anabelle Retondario, Francisco de Assis Guedes de Vasconcelos

**Affiliations:** a Universidade Federal de Santa Catarina, Curitibanos, SC, Brazil.; b Universidade Federal da Fronteira Sul, Realeza, PR, Brazil.; c Universidade Federal de Santa Catarina, Florianópolis, SC, Brazil.; d Universidade de São Paulo, São Paulo, SP, Brazil.

**Keywords:** Breastfeeding, Obesity, Overweight, Child nutrition sciences, Adolescent, Aleitamento materno, Obesidade, Sobrepeso, Ciências da nutrição infantil, Adolescente

## Abstract

**Objective:**

To evaluate the prevalence of breastfeeding (BF) and the association between occurrence/duration of BF and overweight/obesity in schoolchildren aged 7-14 years.

**Methods:**

This is a cross-sectional study, conducted in 2012-2013, on schoolchildren aged 7-14 years from Florianópolis, Santa Catarina, Southern Brazil. Weight and height were measured according to procedures of the World Health Organization. Breastfeeding and sociodemographic data were obtained from a questionnaire responded by parents/guardians. BF was categorized as a dichotomous variable (yes/no) and according to duration (months). Nutritional status was evaluated according to the Z score of the body mass index per age for sex and it was categorized into two groups: normal weight (<Z score+1) and overweight/obesity (≥Z score+1). The adjusted analysis was performed by logistic regression in two age strata (age groups of 7-10 and 11-14 years).

**Results:**

6.6% of schoolchildren had never breastfed; 16.8% had been breastfed for ≤3 months; 16.7%, for 4-6 months; and 59.9%, for ≥7 months. No statistically significant differences were found in the occurrence and duration of BF between the age groups. The prevalence of overweight/obesity was 34.2%. For age groups (7-10 and 11-14 years), the prevalence of overweight/obesity was 36.7% and 29.8%, respectively. Chance of overweight/obesity for the age group of 7-10 years was lower among schoolchildren who were breastfed (OR=0.54; 95%CI 0.33-0.88), when compared with those who never breastfed. When categorized, the chance of overweight/obesity in the age group of 7-10 years was lower for duration of BF ≤3 months (OR=0.41; 95%CI 0.20-0.83), and 4-6 months (OR=0.48; 95%CI 0.28-0.82) when compared with children who never breastfed.

**Conclusions:**

BF for at least six months was associated with a lower chance of overweight/obesity for schoolchildren aged 7-10 years. No association was found for schoolchildren aged 11-14 years.

## RESUMO

**Objetivo:**

Investigar prevalência e duração de aleitamento materno (AM) e sua associação com sobrepeso/obesidade em escolares de 7-14 anos.

**Métodos:**

Estudo transversal, realizado em 2012-2013, com escolares de 7-14 anos de Florianópolis, Santa Catarina. Peso e altura foram mensurados segundo preconizado pela Organização Mundial da Saúde (OMS). Dados sociodemográficos e sobre AM foram obtidos por questionários enviados aos responsáveis/cuidadores. A variável amamentação foi analisada como dicotômica (sim/não) e por duração (em meses). O perfil antropométrico foi avaliado por escore Z do índice de massa corporal (IMC) para a idade, segundo sexo, categorizado em: normal (<escore Z+1) e sobrepreso/obesidade (≥escore Z+1). Análises ajustadas foram realizadas (regressão logística) em dois estratos etários (7-10 e 11-14 anos).

**Resultados:**

6,6% dos escolares nunca tinham sido amamentados, 16,8% foram amamentados por ≤3 meses, 16,7% por 4-6 meses e 59,9% por ≥7 meses. Não houve diferença estatística de ocorrência/duração de AM entre os grupos etários. A prevalência de sobrepeso/obesidade foi 34,2%. Nos grupos etários (7-10 e 11-14 anos), a prevalência foi 36,7% e 29,8%, respectivamente. A chance de sobrepeso/obesidade nos escolares de 7-10 anos foi menor entre aqueles que tinham sido amamentados (OR=0,54; IC95% 0,33-0,88), comparando com os nunca amamentados. Quando categorizada, a chance de ter sobrepeso/obesidade nos escolares de 7-10 anos foi menor quando a duração do AM foi ≤3 meses (OR=0,41; IC95% 0,20-0,83) e 4-6 meses (OR=0,48; IC95% 0,28-0,82), em comparação à ausência de AM.

**Conclusões:**

AM por pelo menos seis meses foi associado com menor chance de sobrepeso/obesidade para escolares de 7-10 anos. Não foi observada associação para o grupo 11-14 anos.

## INTRODUCTION

Obesity is a multifactorial disease associated with biologic and environmental factors.^1^ Among the many associated factors, breastfeeding (BF) seems to be crucial when it comes to such disease.^2^ This evidence has been related to the different composition of breast milk and to its hormonal response when compared with other types of milk, which may also be associated with the adaptation to the diet after BF.^3^ In 2018, Reyes et al. observed that adolescents who were breastfed for less than six months had lower levels of adiponectin, an insulin-sensitizing and cardio-protective hormone that improves the metabolism of lipids and carbohydrates, with anti-inflammatory effects.^4^ A review of this issue, published in 2015, concluded that the neuroendocrine programming could explain the inverse association between BF and overweight/obesity in children.^5^ This review shows that breastmilk has hormonal molecules, such as insulin, insulin-like growth factor I (IGF-1), adiponectin, obestatin, resistin, leptin, and ghrelin, which modulate the development of child fat and lean body mass and the appetite.^5^ However, the hypothesis of this protective effect remains unclear, with some studies conducted in the second half of the 2010s that verified different associations between BF or its duration and obesity.^5^ ^,^ ^6^ ^,^ ^7^

Studies on this association have been carried out in Brazil,^8^ ^,^ ^9^ ^,^ ^10^ particularly on elementary school students, but they were conducted using different methods regarding the definition of outcome and independent variables. Siqueira & Monteiro, in a study conducted in São Paulo in 2007, observed a twice chance of overweight/obesity in children aged 6-14 years who have never been breastfed, compared with those breastfed (dichotomic variable: breastfed *vs.* never breastfed).^8^ In a study carried out in 2007 on schoolchildren from Florianópolis, Southern Brazil, the authors found differences in the association between BF and obesity according to maternal schooling. Schoolchildren aged 7-10 years whose mothers had a lower educational level had a lower chance of obesity if they were breastfed for more than one month. Considering schoolchildren whose mothers have a higher education level, the chance of obesity was 44% lower for those who were breastfed for more than twelve months.^9^ Another study conducted in Florianópolis in 2012/2013 on schoolchildren found a significant association between excess body fat (measured by the skinfold thickness) and exclusive breastfeeding until the sixth month of life.^10^ Studies related to such theme with the same age group conducted in Brazil have shown similar results; however, some associated any period of BF with lower levels of obesity; conversely, others showed an association of a higher duration of BF and outcomes related to obesity in some specific strata (age/maternal schooling).^8^ ^,^ ^9^ ^,^ ^10^

In this context, the objective of this study was to evaluate the association between breastfeeding and breastfeeding duration and overweight/obesity in schoolchildren aged 7-14 years from Florianópolis, Santa Catarina, Brazil, in 2012/2013. This study can contribute to the field of maternal and child nutrition by considering a representative and randomized sample of schools from the municipal district and performing a statistical analysis based on a theoretical model, which allows extrapolating the findings.

## METHOD

This study was performed in Florianópolis, Santa Catarina, Southern Brazil, with a probabilistic sample of schoolchildren aged 7-14 years enrolled in both public and private elementary schools.

The procedures used for calculating the sample size and for the sampling have been previously described.^10^ ^,^ ^11^ Briefly, the sampling universe was 45,247 schoolchildren from 85 public and private schools from Florianópolis, in which we could find classes from all grades of elementary school in the daytime. For sample size, the outcome overweight/obesity was considered according to WHO criteria^12^ (≥Z score+1 body mass index [BMI]-for-age). The expected prevalence of ­overweight/­obesity in this age group, in 2012, would be 38%, according to two cross-sectional studies previously performed in a five-year period and conducted in Florianópolis in 2002^13^ and 2007,^14^ with the same study population, which found prevalence of 30 and 34%, respectively. Considering a sampling error of 3.5 percentage points (two-sided), and a 95% confidence interval, a sample size of 727 schoolchildren was achieved. When applying a design effect (DEFF) of 1.8, based on the aforementioned research conducted in 2007, the sample size would comprise 1,309 schoolchildren. However, considering the aim to compare the findings of the present study with the results from the two studies previously conducted in the city, by stratifying the sample by age group (7-10 and 11-14 years of age), the sample size was duplicated. Finally, a 10% safety margin was added for potential losses and/or individuals refusing to participate in the study, thus comprising a final sample size of 2,880 schoolchildren.

The present study includes a probabilistic sample of 2,506 schoolchildren, stratified by municipal administrative districts, type of school (public or private), and age group (7-10 and 11-14 years). A total of 30 schools were randomly selected (19 public and 11 private schools). Schoolchildren were selected from each school by clusters.

This study was conducted according to the guidelines established in the Declaration of Helsinki and was approved in 2012 by the Committee of Ethics in Research with Human Beings from Universidade Federal de Santa Catarina (no. 120341/2012), according to the standards established by Resolution no. 466/2012 of the National Health Council. Students who had the permission of their parents or guardians to participate in the research were included by signing the informed consent form.

Data were collected from September/2012 to June/2013. The team responsible for data collection was trained by a researcher certified by the International Society for the Advancement of Kinanthropometry, and a pilot study for anthropometric standardization was carried out in a school that was not chosen to compose the sample. The absolute intra-examiner Technical Error of Measurement (TEM) deemed acceptable was twice that of the certified researcher, whereas the absolute inter-examiner TEM deemed acceptable was thrice that of the certified researcher. Anthropometric measurements were performed according to the World Health Organization (WHO).^12^ Body weight was measured using an electronic Marte^®^ scale (180 kg of capacity; 50 g of accuracy); height was measured using an Alturexata^®^ stadiometer (1 mm of accuracy).

Data related to BF and other information used as control variables were obtained from a questionnaire sent to the ­parents/­guardians (96.1% of questionnaires were answered by mothers or fathers). Data on the schoolchildren’s birth and childhood (birth weight, gestational age, and BF duration), demographic data (maternal schooling and age, total monthly income, number of household members) as well as anthropometric data related to their mothers (self-reported weight and height) were collected. Covariate sexual maturation was collected from students aged 11-14 years. The schoolchildren self-reported their stage through a spreadsheet containing figures corresponding to the sexual maturation stages.

BF data were obtained based on the following question to the parents/guardians: “Was the student breastfed?” “If yes, for how long?’’. This question was divided into BF time categories, which were grouped for data analysis.

Schoolchildren’s nutritional status (outcome variable) was categorized into two groups: normal weight (<Z score+1) and overweight/obesity (≥Z score+1).^12^

BF (exposure variable) was analyzed in two different ways: first, it was dichotomously categorized (yes *vs*. no) as ‘‘had never been breastfed (no)’’ or ‘‘had been breastfed for some period (yes)’’; then, it was categorized according to the BF duration: ‘‘had never been breastfed,” “had been breastfed for up to 3 months,” “had been breastfed from 4 to 6 months,” or “had been breastfed for ≥7 months.”

Obesity diagnosis in adolescence is established by body composition related to sex and sexual maturation. Considering that the relationship between BMI and body fat depends on sexual maturation,^15^ the schoolchildren’s age was classified into two groups: 7-10 years and 11-14 years, in order to characterize children and adolescents. Taking this influence into consideration, the association analysis was separately performed according to the age group.

The independent variables considered in this study include: sex, maternal age (20-29 years; 30-39 years; ≥40 years), maternal schooling (0-8 years; 9-11 years; ≥12 years), maternal nutritional status (weight and height were self-reported and Body Mass Index was calculated and classified into underweight/normal weight, BMI<25.0 kg/m^2^; pre-obesity, BMI=25.0-29.9 ­kg/­m^2^; and obesity, BMI≥30 kg/m^2^, according to WHO);^12^ type of school (public; private), *per capita* family income (divided in tertiles), birth weight (considered as low/insufficient when birth weight ≤2999 g), sexual maturation for 11-14-year-old students (normal - pubic hair and breast/genital growth in the same stage of development; early - pubic hair one or two stages ahead of breast/genital development; late - pubic hair one or two stages behind breast/genital development);^16^ and gestational age at the student’s birth (premature if gestational age<37 weeks).

**Figure 1:**
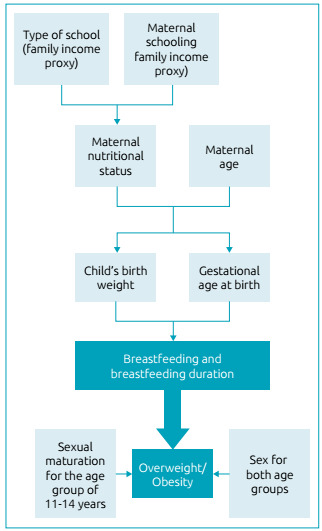
Theoretical analysis model for the development of overweight/obesity.

Data were entered twice and processed by the EpiData^®^ 3.0 software. All data were verified and automatically checked for consistency and amplitude purposes. Analyses were performed using the STATA^®^ 15.0 statistical software (StataCorp, Texas, USA). Data from each age group were analyzed according to maternal and child characteristics and the nutritional status using the chi-square test and 95% confidence interval. Logistic regression was used for crude and adjusted analysis. The theoretical analysis model presented in Figure 1 shows the relationships among these variables and how they can influence the outcomes.^6^ ^,^ ^8^ ^,^ ^17^ ^,^ ^18^ Maternal nutritional status is influenced by socioeconomic characteristics and, in its turn, it influences (together with maternal age) the child’s birth. Gestational age at birth and child’s birth weight influence the chance of children being breastfed and, if so, for how long they have been. Breastfeeding, in turn, influences overweight/obesity, together with sex and sexual maturation, for children aging over 11 years.^6^ ^,^ ^8^ ^,^ ^17^ ^,^ ^18^ This model based the choice of variables on what would be tested in the crude analysis. Exposure variables with p≤0.25 for crude associations were entered into the adjusted model. Adjustment in the age group of 7-10 years was made by the variables sex, type of school, mother’s age, maternal nutritional status, child birth weight, and maternal schooling; and the adjustment for schoolchildren aged 11-14 years was made by sex, type of school, mother’s age, maternal nutritional status, child sexual maturation, and maternal schooling. Maternal schooling was considered instead of the income, because it is an indicator of socioeconomic status more stable over time than income.^19^ In addition, the type of school was used because, in Brazil, children who attended private schools probably experienced better socioeconomic conditions than those who attended public schools.^20^ The Survey (SVY) command from STATA^®^ was used in order to consider sampling effect. All regression results are shown as *Odds Ratio* (OR) with their respective 95% confidence intervals (95%CI). Variables with p<0.05, or when there was no overlap of the 95%CI, were considered statistically significant.

## RESULTS

A total of 4,082 schoolchildren aged 7-14 years were considered eligible to this study. The final study sample consisted of 2,506 schoolchildren, with 1,531 aged 7-10 years and 975, 11-14 years, considering the 61.4% adherence rate (38.6% of losses/refusals). Comparisons between evaluated and not-evaluated (loss or/and refusal) schoolchildren aged 7-10 years in this study showed a higher percentage of boys, schoolchildren enrolled in private schools, and those attending the early grades among not-evaluated schoolchildren. As for adolescents, the prevalence of boys, enrolled in public schools and aged 13-14 years (when compared with adolescents aged 11-12 years) were higher among those not-evaluated.

Regarding BF, 6.6% had never been breastfed; 16.8% had been breastfed for up to three months; 16.7%, from four to six months (95%CI 14.6-19.0%); and 59.9%, for seven months or more (95%CI 56.8-62.9%). No differences were found in the prevalence related to the occurrence of BF and its duration when comparing the groups (p=0.728 and p=0.508, respectively). Expected differences were observed regarding maternal age for schoolchildren in the age group of 11-14 years when compared with younger schoolchildren (p<0.001). There was a higher proportion of mothers with less education in the age group of 11-14 years, but it was not statistically significant (p=0.120), and children were born preterm in the same age group compared with schoolchildren from the age group of 7-10 years (p=0.004). Regarding ­overweight/­obesity, the prevalence for the total sample was 34.2%; for schoolchildren aged 7-10 years, 36.7%; and for adolescents aged 11-14 years, 29.8%. There was a statistically significant difference between the prevalence of ­overweight/­obesity in both groups (p=0.024), but the confidence intervals overlapped (Tables 1 and 2).

**Table 1:** Description of the sample of schoolchildren from the age group of 7-14 years. Florianópolis, SC, Brazil, 2012/2013.

Characteristics	Total (n=2,506)		7-10 years (n=1,531; 61%)		11-14 years (n=975; 39%)		p-value*
	n (%)	95%CI	n (%)	95%CI	n (%)	95%CI	
Student’s sex (2,506)							
Male	1,090 (43.5)	40.4-46.6	687 (44.9)	40.9-49.0	404 (41.1)	36.7-45.7	0.188
Female	1,416 (56.5)	53.4-59.6	844 (55.1)	51.0-59.1	571 (58.9)	54.3-63.3	
Per capita income (2,152)**							
1^st^ tertile	676 (31.4)	26.5-36.8	404 (30.5)	24.7-36.9	273 (33.0)	27.8-38.8	0.669
2^nd^ tertile	740 (34.4)	28.1-41.2	466 (35.2)	27.7-43.5	273 (33.0)	27.2-39.2	
3^rd^ tertile	736 (34.2)	25.5-44.2	454 (34.3)	23.5-47.2	282 (34.0)	27.3-41.4	
Birth weight ≤2999 g (2,335)							
Yes	670 (28.7)	26.4-31.1	423 (29.2)	26.2-32.3	247 (27.8)	24.3-31.7	0.573
No	1,665 (71.3)	68.9-73.6	1025 (70.8)	67.7-73.8	640 (72.2)	68.3-75.7	
Gestational age <37 weeks (2,238)							
Yes	461 (20.6)	18.6-22.8	254 (18.3)	16.1-20.8	209 (24.7)	21.2-28.5	0.004
No	1,777 (79.4)	77.2-81.4	1136 (81.6)	79.2-83.9	639 (75.3)	71.5-78.8	
Type of school (2,506)							
Public	1591 (63.5)	52.9-72.9	934 (61.0)	48.7-72.0	661 (67.8)	57.9-76.3	0.173
Private	915 (36.5)	27.1-47.1	597 (39.0)	28.0-51.3	314 (32.2)	23.7-42.1	
Maternal breastfeeding (2,423)							
No	160 (6.6)	5.1-8.4	102 (6.8)	4.9-9.4	58 (6.2)	4.1-9.1	0.728
Yes	2,263 (93.4)	91.6-94.9	1393 (93.2)	90.6-95.1	870 (93.8)	90.9-95.9	
Breastfeeding duration (2,423)							
Never breastfed	160 (6.6)	5.1-8.4	102 (6.8)	4.9-9.4	58 (6.2)	4.1-9.1	0.508
≤3 months	407 (16.8)	14.6-19.3	234 (15.7)	13.0-18.9	175 (18.9)	15.9-22.2	
4 to 6 months	405 (16.7)	14.6-19.0	252 (16.9)	14.6-19.4	152 (16.4)	13.3-20.1	
≥7 months	1,451 (59.9)	56.8-62.9	907 (60.6)	57.1-64.1	543 (58.5)	53.8-63.1	
Nutritional status of schoolchildren (2,484)							
Normal weight	1,634 (65.8)	63.5-68.1	963 (63.3)	59.9-66.5	675 (70.2)	65.9-74.1	0.024
Overweight/obesity	850 (34.2)	31.9-36.5	559 (36.7)	33.5-40.1	287 (29.8)	25.9-34.1	
Sexual maturation (952)							
Early	34 (3.6)	22.9-33.4	-	-	34 (3.6)	1.3-9.9	-
Normal	824 (86.6)	64.9-70.8	-	-	824 (86.6)	84.5-88.4	
Late	94 (9.8)	1.9-9.2	-	-	94 (9.8)	6.4-14.8	

*Chi-square test; **1^st^ tertile: median=BRL 1,200.00; 2^nd^ tertile: median=BRL 2,000.00; 3^rd^ tertile: median=BRL 5,200.00. Data not collected for the age group of 7-10 years; 95%CI: 95% confidence interval.

**Table 2:** Description of maternal characteristics regarding schoolchildren from the age group of 7-14 years. Florianópolis, SC, Brazil, 2012/2013.

Characteristics	Total (n=2,506)		7-10 years (n=1,531; 61%)		11-14 years (n=975; 39%)		p-value*
	n (%)	95%CI	n (%)	95%CI	n (%)	95%CI	
Maternal age (2,325)							
20-29 years	335 (14.4)	11.9-17.4	268 (18.7)	14.8-23.4	61 (6.9)	5.2-9.0	<0.001
30-39 years	1,153 (49.6)	46.6-52.6	729 (50.8)	47.4-54.2	424 (47.6)	41.7-53.5	
≥40 years	837 (36.0)	32.0-40.1	438 (30.5)	26.4-34.8	405 (45.5)	39.3-52.0	
Maternal schooling (2,389)							
0-8 years	317 (24.6)	5.6-64.3	326 (22.1)	4.2-64.6	264 (29.0)	8.4-64.6	0.120
9-11 years	865 (36.2)	17.8-59.7	526 (35.6)	16.4-60.9	339 (37.2)	20.3-58.0	
≥12 years	1,207 (39.3)	6.4-85.9	625 (42.3)	7.0-87.7	309 (33.8)	5.3-82.5	
Maternal nutritional status (2,286)							
Underweight/normal weight	1,344 (56.8)	52.4-61.0	829 (58.2)	53.2-63.1	467 (54.2)	49.2-59.1	0.198
Pre-obesity	640 (28.0)	24.8-31.5	397 (27.9)	24.0-32.0	244 (28.3)	24.2-32.9	
Obesity	302 (15.2)	12.9-17.8	198 (13.9)	11.2-17.1	151 (17.5)	13.9-21.8	

*Chi-square test; 95%CI: 95% confidence interval.

The association between schoolchildren’s characteristics and the nutritional status stratified by age groups is demonstrated in Table 3. In both age groups, a higher proportion of pre-obese and obese mothers was observed in ­overweight/­obesity schoolchildren when compared with those without ­overweight/­obesity (p<0.001). Concerning children aged 7-10 years, a greater proportion of children with sufficient birth weight were overweight/obese when compared with those with normal weight (p=0.004). A higher proportion of overweight/obesity was observed in children attending public schools (p=0.038). In adolescents, the variables associated with overweight/obesity were sex and maternal age (p=0.027 and p=0.008, respectively).

**Table 3:** Association between nutritional status and characteristics of the sample stratified by age group. Florianópolis, SC, Brazil, 2012/2013.

Characteristics	7-10 years (n=1,531)				11-14 years (n=975)			
	Normal weight		Overweight/Obesity		Normal weight		Overweight/Obesity	
	n (%)	95%CI	n (%)	95%CI	n (%)	95%CI	n (%)	95%CI
Student’s sex (2,506)								
Male	414 (42.5)	34.2-51.1	269 (49.2)	45.7-52.7	261 (38.2)	27.9-49.6	133 (47.8)	38.4-57.4
Female	561 (57.5)	48.8-65.8	278 (50.8)	47.3-54.3	422 (61.9)	50.4-72.1	146 (52.2)	42.6-61.6
p-value*	0.054				0.027			
Maternal age (2,325)								
20-29 years	178 (19.5)	6.7-44.9	87 (16.9)	5.4-42.3	53 (8.6)	3.2-21.5	7 (2.6)	0.9-7.4
30-39 years	468 (51.2)	48.8-58.7	257 (50.1)	39.0-61.3	277 (44.7)	26.5-64.5	141 (54.2)	35.2-72.1
≥40 years	268 (29.3)	12.0-55.6	168 (32.9)	24.8-42.3	289 (46.6)	22.3-72.7	113 (43.1)	24.6-63.7
p-value*	0.404				0.008			
Maternal schooling (2,389)								
0-8 years	218 (23.2)	4.9-64.0	105 (19.8)	3.2-65.1	168 (26.6)	7.9-60.3	91 (33.8)	8.7-73.4
9-11 years	346 (36.9)	17.6-61.5	178 (33.6)	14.2-60.9	241 (38.1)	19.0-61.7	95 (35.5)	19.4-55.7
>12 years	374 (39.9)	6.4-86.6	247 (46.5)	8.1-89.6	224 (35.4)	5.6-83.4	82 (30.7)	4.5-80.7
p-value*	0.065				0.246			
Maternal nutritional status (2,286)								
Underweight/Normal weight	603 (66.5)	42.2-84.3	226 (44.5)	26.5-64.1	370 (61.8)	39.0-80.3	94 (37.1)	25.3-50.7
Pre-obesity	199 (21.9)	13.0-34.7	194 (38.1)	22.9-56.0	152 (25.4)	14.5-40.6	89 (35.2)	20.4-53.5
Obesity	105 (11.6)	3.6-31.6	88 (17.4)	13.7-21.9	77 (12.8)	3.9-34.7	71 (27.7)	12.4-50.8
p-value*	< 0.001				< 0.001			
Per capita income (2,152)								
1^st^ tertile	273 (32.7)	8.5-71.6	129 (26.8)	5.8-68.7	185 (32.2)	9.5-68.2	82 (33.6)	9.6-70.6
2^nd^ tertile	290 (34.7)	15.9-59.8	173 (36.0)	14.7-64.7	193 (33.6)	15.9-57.5	78 (31.9)	20.4-46.2
3^rd^ tertile	273 (32.7)	31.5-87.9	179 (37.2)	4.1-89.0	195 (34.2)	4.6-85.0	83 (34.5)	6.0-81.2
p-value*	0.127				0.921			
Birth weight ≤2999g (2,335)								
Yes	304 (33.1)	28.7-37.9	117 (22.5)	13.4-35.3	165 (26.7)	24.6-28.9	79 (30.8)	20.2-43.8
No	613 (66.9)	62.1-71.3	405 (77.5)	64.7-86.6	453 (73.3)	71.1-75.4	178 (69.3)	56.2-79.8
p-value*	0.004				0.336			
Gestational age <37 weeks (2,238)								
Yes	160 (18.1)	12.3-26.0	92 (18.4)	9.8-31.8	141 (24.3)	17.5-32.7	64 (25.2)	22.9-27.7
No	723 (81.9)	74.0-87.7	407 (81.6)	68.2-90.2	441 (75.7)	67.3-82.5	190 (74.8)	72.3-77.1
p-value*	0.942				0.813			
Type of school (2,506)								
Public	618 (63.4)	3.7-98.7	310 (56.7)	2.6-98.5	455 (66.6)	4.5-98.8	196 (70.4)	4.7-99.1
Private	357 (36.6)	12.7-96.3	237 (43.3)	1.5-97.4	228 (33.4)	1.2-95.5	83 (29.6)	0.9-95.3
p-value*	0.038				0.440			
Maternal breastfeeding (2,423)								
No	53 (5.6)	3.6-8.6	47 (8.8)	3.6-19.8	41 (6.4)	2.9-13.3	15 (5.7)	2.5-12.2
Yes	897 (94.4)	91.4-96.4	489 (91.3)	80.2-96.4	603 (93.6)	86.7-97.1	256 (94.3)	87.8-97.5
p-value*	0.058				0-678			
Breastfeeding duration (2,423)								
Never breastfed	53 (5.6)	3.6-8.6	47 (8.8)	3.6-19.8	41 (6.4)	2.9-13.3	15 (5.7)	2.5-12.2
≤3 months	161 (16.9)	10.6-25.7	74 (13.8)	10.0-18.7	120 (18.7)	11.8-28.3	54 (19.9)	13.1-29.0
4 to 6 months	174 (18.3)	14.7-22.6	78 (14.5)	11.7-17.8	105 (16.3)	14.4-14.8	45 (16.5)	10.7-24.7
≥7 months	388 (59.3)	55.7-62.7	337 (62.9)	53.2-71.7	378 (58.6)	55.5-61.7	157 (57.9)	44.5-70.2
p-value*	0.086				0.960			
Sexual maturation (952)								
Early	-	-	-	-	30 (4.4)	1.5.12.1	5 (1.8)	0.2-17.3
Normal	-	-	-	-	592 (87.7)	85.3-89.8	230 (83.9)	82.7-85.0
Late	-	-	-	-	53 (7.9)	4.7-12.4	39 (14.2)	9.7-20.5
p-value*	-				0.118			

*Chi-square test. Data not collected for the age group of 7-10 years; 95%CI: 95% confidence interval.

The prevalence of overweight/obesity, crude and adjusted OR, according to BF and BF duration categories for the age group of 7-10 years is demonstrated in Table 4. Lower chance of overweight/obesity was observed in children who were breastfed (OR=0.54; 95%CI 0.33-0.88). This association was maintained in the categories of breastfeeding duration ≤3 months (OR=0.41; 95%CI 0.20-0.83) and 4-6 months (OR=0.48; 95%CI 0.28-0.82) when compared with schoolchildren who had never been breastfed in this age stratum. Data on the age group of 11-14 years are demonstrated in Table 5. No association was found between BF or BF duration and ­overweight/­obesity in adolescents.

**Table 4:** Prevalence (%) of overweight/obesity*, crude and adjusted *Odds Ratio* according to maternal breastfeeding and breastfeeding duration for schoolchildren from the age group of 7-10 years. Florianópolis, SC, Brazil, 2012/2013.

Characteristics**	Crude OR# (95%CI)	Adjusted OR#1 (95%CI)	Adjusted OR#2 (95%CI)
Maternal breastfeeding (1,486)			
Yes	0.62 (0.36-1.02)	0.54 (0.33-0.88)	-
Breastfeeding duration (1,486)			
≤3 months	0.52 (0.27-0.98)	-	0.41 (0.20-0.83)
4 to 6 months	0.51 (0.29-0.89)	-	0.48 (0.28-0.82)
≥7 months	0.68 (0.41-1.31)	-	0.61 (0.37-1.00)
Student’s sex (1,522)			
Female	0.76 (0.58-1.00)	0.70 (0.49-0.99)	0.70 (0.49-0.97)
Maternal age (1,427)			
30-39 years	1.13 (0.80-1.58)	0.88 (0.62-1.25)	0.87 (0.62-1.24)
≥40 years	1.29 (0.93-1.80)	1.02 (0.70-1.49)	1.00 (0.68-1.47)
Maternal schooling (1,468)			
9-11 years	1.07 (0.74-1.53)	1.14 (0.74-1.73)	1.15 (0.75-1.76)
≥12 years	1.36 (0.94-1.99)	1.46 (0.86-2.48)	1.53 (0.88-2.67)
Maternal nutritional status (1,415)			
Pre-obesity	2.59 (1.95-3.44)	2.93 (2.15-3.99)	2.98 (2.17-4.09)
Obesity	2.24 (1.37-3.67)	2.40 (1.36-4.25)	2.51 (1.46-4.32)
Birth weight ≤2999 g (1,439)			
Yes	0.59 (0.42-0.82)	0.58 (0.41-0.84)	0.58 (0.42-0.85)
Gestational age <37 weeks (1,382)			
Yes	1.02 (0.65-1.60)	-	-
Type of school (1,522)			
Private	1.32 (1.02-1.71)	0.58 (0.41-0.84)	1.29 (0.89-1.25)

*Overweight/obesity: body mass index (BMI) Z score≥1; **adjusted for sex (reference: male), maternal age (reference: 20-29 years), maternal schooling (reference: 0-8 years), mother’s nutritional status (reference: underweight/normal weight), birth weight (reference: normal birth weight), and type of school (reference: public school); #1Normal weight as reference group. OR: *Odds Ratio*; 95%CI: 95% confidence interval. Values for the main exposure variable “Maternal breastfeeding”; #2Normal weight as reference group. Values for the main exposure variable “Breastfeeding duration.”

**Table 5:** Prevalence (%) of overweight/obesity*, crude and adjusted *Odds Ratio* according to maternal breastfeeding and breastfeeding duration for schoolchildren from the age group of 11-14 years. Florianópolis, SC, Brazil, 2012/2013.

Characteristics**	Crude OR# (95%CI)	Adjusted OR#1 (95%CI)	Adjusted OR#2 (95%CI)
Maternal breastfeeding (915)			
Yes	1.13 (0.61-2.09)	1.63 (0.70-3.78)	-
Breastfeeding duration (915)			
≤3 months	1.20 (0.60-2.37)	-	1.85 (0.78-4.39)
4 to 6 months	1.14 (0.51-2.54)	-	1.67 (0.61-4.60)
≥7 months	1.11 (0.60-2.04)	-	1.54 (0.65-3.64)
Student’s sex (962)			
Female	0.67 (0.48-0.94)	0.67 (0.47-0.95)	0.67 (0.47-0.96)
Maternal age (880)			
30-39 years	3.91 (1.61-9.47)	3.13 (1.42-7.75)	3.26 (1.39-7.62)
≥40 years	2.98 (1.17-7.59)	2.61 (1.07-6.35)	2.63 (1.08-6.37)
Maternal schooling (901)			
9-11 years	0.73 (0.40-1.34)	0.71 (0.39-1.28)	0.72 (0.40-1.29)
≥12 years	0.68 (0.36-1.29)	0.87 (0.42-1.80)	0.88 (0.42-1.84)
Maternal nutritional status (853)			
Pre-obesity	2.31 (1.47-3.62)	2.09 (1.34-3.27)	2.12 (1.35-3.33)
Obesity	3.60 (2.05-6.31)	3.65 (2.01-6.63)	3.65 (2.00-6.62)
Birth weight ≤2999 g (875)			
Yes	1.22 (0.82-1.80)	-	-
Gestational age <37 weeks (836)			
Yes	1.05 (0.69-1.60)	-	-
Type of school (962)			
Private	0.84 (0.53-1.33)	1.11 (0.68-1.80)	1.11 (0.69-1.79)
Sexual maturation (949)			
Normal	2.28 (0.81-6.37)	2.07 (0.62-6.90)	2.11 (0.63-7.02)
Late	4.31 (1.48-12.54)	3.82 (1.6-12.54)	3.93 (1.21-12.80)

*Overweight/obesity: body mass index (BMI) Z score≥1; **adjusted for sex (reference: male), maternal age (reference: 20-29 years), maternal schooling (reference: 0-8 years), mother’s nutritional status (reference: underweight/normal weight), type of school (reference: public school), and sexual maturation (reference: early); #1Normal weight as reference group. OR: *Odds Ratio*; 95%CI: 95% confidence interval. Values for the main exposure variable “Maternal breastfeeding”; #2Normal weight as reference group. Values for the main exposure variable “Breastfeeding duration.”

## DISCUSSION

This study aimed to evaluate the prevalence of schoolchildren’s breastfeeding and their characteristics as well the association between BF and BF duration with ­overweight/­obesity. We found 6.6% of schoolchildren who have never been breastfed, 33.5% who were breastfed for at least six months, and a proportion of almost 60% who were breastfed for seven months or more. Furthermore, for the youngest group (7-10 years of age) - and after adjusting the analyses for maternal and socioeconomic influences - there were lower chance of overweight/obesity among schoolchildren breastfed compared with those who had never been breastfed. Moreover, still concerning the youngest group, being breastfed for at least 3 or 4-6 months showed the same statistically inverse association with overweight/obesity. No association was found for BF and overweight/obesity in schoolchildren aged 11-14 years.

Concerning the prevalence of breastfed children, the National Survey on Breastfeeding Prevalence, conducted in 2008, found that 58.7% of 9-12-month children had been breastfed in Brazil - 52.2% in the city of Florianópolis.^21^ When comparing this fact with data from other countries, Rossiter et al., in 2011, developed a cross-sectional study on a population of 5,560 schoolchildren aged 10-11 years in Nova Scotia (Canada), and found that 32% of children were breastfed for at least six months, a similar prevalence compared with the one found in this study (33.5%).^22^ The WHO and the Brazilian Ministry of Health recommend exclusive BF for children younger than six months and the maintenance of BF with appropriate complementary feeding until they turn at least two years old.^23^ In the present study, 59.9% of 7-14-year-old children had been breastfed for more than six months. However, 6.6% of both groups had not been breastfed for any period. This indicates that actions aimed at encouraging BF that are performed in Brazil since the end of the 1980s should be maintained. Among actions developed in the period of this study, the *Estratégia Amamenta e Alimenta Brasil* [Feeding and Breastfeeding Brazil Strategy] can be mentioned, which is an initiative launched in the 2013 aimed at qualifying human resources in public healthcare units to promote, protect, and support breastfeeding and healthy complementary feeding.^23^ Examples of recommended actions on behalf of this strategy are to properly welcome mothers and their newborn child in the healthcare units and to establish a continuous tie between the health professionals and the mothers and their families. This issue is very important, as observed by Zakarija-Grković et al. in 2015, in a study conducted in Croatia on 773 mothers. The authors found that the chance of being exclusively breastfed until six months of age was 2.6 higher if mothers had a nutritional counseling on infant feeding with a health professional before childbirth.^24^

Regarding the prevalence of overweight/obesity in this study (36.7% among younger children and 29.8% among adolescents), it is in accordance with the Brazilian National Dietary Survey 2008-2009 (POF, Portuguese acronym for *Pesquisa de Orçamentos Familiares*, 2008/2009), in which ­overweight/­obesity prevalence was higher among younger groups (48.2% in children aged 7-9 years, and 30.0% in those aged 10-15 years).^25^ In a cohort study conducted by Parrino et al. on 1,521 Sicilian children aged 9-14 years, the prevalence of obesity was 14.1%.^26^ It is worth noting that childhood and adolescence obesity is a risk factor for obesity in adulthood.^7^

Results from adjusted analyses in the present study showed a statistical association between maternal BF and ­overweight/­obesity prevalence among schoolchildren aged 7-10 years, and between BF duration (≤ 6 months) and overweight/obesity prevalence among schoolchildren aged 7-10 years; however, such association was not found in the older group (schoolchildren aged 11-14 years). The results of this study are similar to the findings of Grube et al., who conducted a study on German children and adolescents in 2003-2006 and observed a protective effect of BF on children aged 7-10 years, although they did not find an association in other age groups (3-6, 11-13, and 14-17 years of age).^27^ While the results of the present study are only significant among younger schoolchildren, another Brazilian study conducted in 2007 on students aged 6-14 years found twice the chance of obesity in schoolchildren who had never been breastfed.^9^ On the other hand, a study conducted by Brion et al., with data from a Brazilian cohort born in 1993, found no relationship between BF and obesity at the age of nine.^8^

Different results from the aforementioned studies can be explained by their distinct confounding variables inserted in the final analysis model. In a systematic review, Horta et al. observed that BF is related to lower rates of obesity and overweight in general. However, they identified that studies in which estimates were adjusted for confounding variables, such as socioeconomic status, birth condition, and parental anthropometry, found a poorer association between BF and risk of obesity in later life (this review included studies on children and adults) when compared with those that did not consider it.^6^ The study conducted in Germany evaluates the effect of BF on the nutritional status of children and adolescents, and have found a similar decrease in the association when taking adjusted variables into consideration.^27^ Although studies have documented an association between breastfeeding and reduction in the risk of child obesity, there are unobservable confounders that may influence correlations between breastfeeding behaviours and child weight outcomes. However, the International Early Nutrition Research Project has summarized, by a systematic review, that there is an inverse association between BF and overweight/obesity in childhood and adulthood (13% decreased odds).^28^ Nonetheless, the authors reinforced that they cannot exclude residual confounding from this association.^28^

In this study, an association between schoolchildren aged 7-10 years and the lack of association between the oldest age group have already been verified in the crude analysis for BF duration and, therefore, have remained after the inclusion of different variables in the adjusted model. Concerning potential effect modifiers, it should be highlighted that a previous study with similar design has been conducted in Florianópolis in 2007. The authors showed that chances of obesity may vary according to BF duration with a modifying effect of the maternal schooling. Moreover, in the previous study, for younger children whose mothers had lower schooling level (0-8 years), the chance of obesity was lower for any period of breastfeeding longer than one month.^10^ When analyzing the most recent study, BF has not played a more important role in older children when it comes to overweight/obesity prevention. Perhaps the influence of other variables, such as food consumption, physical activity - which has been changing over time in this age group^25^ -, in addition to maternal-related influences, may play a more important role in nutritional status in adulthood, considering that maternal weight status could influence the overall variability of BMI.^20^

The primary limitation of this study is related to its cross-sectional design, in such a way that additional evidence is needed to support its findings. Even though questions on BF duration were retrospectively assessed, thus being subject to memory bias, this is a common procedure often carried out in other studies, and it has been considered a valid and reliable estimate of BF onset and duration.^29^ ^,^ ^30^ In addition, some variables, such as maternal weight and height for estimating BMI, were self-reported. Finally, data on exclusive BF, complementary feeding, and utilization of infant formula were not included.

The representative and randomized sample of schools from all the five geographical regions in the relevant municipal district is one of the strengths of this study. Therefore, the results can be extrapolated from the population of schoolchildren aged 7-14 years from the municipality of Florianópolis, Brazil. The weighting effect of each person on the sample was also considered, which minimizes bias in the analysis of the variables, for which fewer responses were found.

In conclusion, BF was associated with overweight/obesity concerning schoolchildren aged 7-10 years living in Florianópolis, Southern Brazil. Besides the confirmation of such findings by longitudinal studies, it is highly recommended for Brazilian authorities to create more actions and programs for the National Food and Nutrition Policy on breastfeeding, which seems to be crucial for the early development of overweight and obesity.
